# Exploring the Impact of Sociodemographic Characteristics and Health Literacy on Adherence to Dietary Recommendations and Food Literacy

**DOI:** 10.3390/nu15132853

**Published:** 2023-06-23

**Authors:** Alina Ioana Forray, Mădălina Adina Coman, Răzvan Mircea Cherecheș, Cristina Maria Borzan

**Affiliations:** 1 Discipline of Public Health and Management, Department of Community Medicine, Iuliu Hațieganu University of Medicine and Pharmacy, 400349 Cluj-Napoca, Romania; 2 Department of Public Health, Faculty of Political, Administrative and Communication Sciences, Babeș-Bolyai University, 400132 Cluj-Napoca, Romania; madalina.coman@publichealth.ro (M.A.C.);

**Keywords:** health literacy, dietary recommendations, dietary intake, food literacy, self-rated health, adherence, Romania, representative sample, cross-sectional study

## Abstract

This study investigates food literacy-related abilities and adherence to dietary recommendations in relation to sociodemographic characteristics and health-related features (health literacy, self-rated health and morbidity) in the North-Western region of Romania. This is a secondary analysis of cross-sectional data collected in 2019 from a representative and randomised sample of 1572 individuals. A questionnaire was employed to record participants’ sociodemographic characteristics, food-related and health-related features. Most participants were non-adherent to dietary recommendations for fruit and vegetables (83.5%), fish and seafood (61.3%), and water intake (67.9%). However, most participants reported an adequate ability to understand the connection between nutrition and health (89.1%), to distinguish between healthy and less healthy options (84.4%), and to acquire nutrition information (75.6%). Non-adherence to dietary recommendations and low food literacy abilities were more prevalent in disadvantaged groups (older age, rural settings, retirement or social welfare, low educational attainment, formerly married). Health literacy was negatively associated with not adhering to dietary recommendations and poor self-rated food literacy abilities. The study suggests that low socioeconomic status negatively impacts food literacy and adherence to dietary recommendations among Romanian adults. Identifying target populations to improve food-related abilities and health literacy can aid public health services in improving health outcomes.

## 1. Introduction

The relationship between nutrition and disease is well-researched. Evidence is available to help develop solutions to prevent nutrition-related diseases and implement large-scale options, especially in key populations [1,2,3]. Health literacy (HL) is a mediator of health outcomes, necessary for understanding and using common health information. Deficiencies in health education are associated with low access to preventive care services, difficulties with self-management of chronic diseases, and inadequate health status of populations [4]. Increasing scientific evidence demonstrates that those with low health literacy suffer from chronic non-communicable diseases in a higher proportion and show adverse health outcomes [5,6]. Since nutrition is a major fundamental factor in developing and treating type 2 diabetes mellitus, high blood pressure, hyperlipidemia, and obesity, reduced food literacy (FL) can be particularly alarming. Improved HL and FL have been shown to result in diets that are both healthier and of higher quality [7,8]. Similarly, diets of higher quality have been linked to a lower risk of developing chronic diseases related to diet [9]. Preventing these diseases in any population requires understanding and applying nutritional knowledge, referred to as the nutritional awareness [10].

Although eating habits are multifactorial, FL represents an important determining factor [11,12]. FL, an integral component of health literacy (HL), is a collection of interrelated knowledge, skills, and behaviours critical for a healthy diet. Specifically, FL encompasses the four domains of planning, managing, selecting, preparing, and eating, providing a framework for understanding and influencing dietary behaviours. Existing FL interventions are highly varied in nature, targeting different groups, implementing various strategies, and assessing different FL domains [13]. Given the broad cultural, social, and economic factors that shape food and diet choices, such interventions must be carefully tailored to specific population groups [14,15]. In addition to this demographic diversity, successful interventions must account for the diverse dietary needs across different developmental stages of a person’s lifespan [16]. Within this context, FL involves more than just acquiring specific skills. It requires understanding the health-related consequences, such as overweight and obesity, and developing effective learning mechanisms, such as information seeking and professional support. This comprehensive view of FL implies that these factors are not merely part of FL’s definition but are influential elements that are both affected by and can influence FL [17]. Although the significance of FL is increasingly recognised, research on FL in Europe, including Romania, is scarce [18]. There is a critical need for evidence-based research providing a comprehensive understanding of FL’s domains, determinants, and influential factors. Such research could help develop effective food-related knowledge, behaviours, skills, and systems [17].

Previous research typically focused on the associations between dietary behaviours, HL, FL, and quality of life in populations with certain chronic conditions [19,20,21,22,23,24]. A systematic review from 2018 evaluated the association between health literacy and adherence to dietary recommendations [25]. It produced mixed results, with only five out of eleven associations between health literacy and eating practices being significant and direct. The authors suggested that research on how health literacy relates to dietary adherence is limited, and further studies on the subject should be conducted. Adhering to dietary guidelines is linked to improved health outcomes. Therefore, people are encouraged to improve their diet by reducing their consumption of certain foods and increasing their consumption of healthy options such as fruits and vegetables [26].

In this regard, little is known about FL and dietary behaviours in Romania. There is a strong need for more accurate data and effective nutrition programs in Romania, a country still facing problems of undernutrition and in which issues associated with food overconsumption and diet-related non-communicable diseases are on the rise [27,28,29]. Recent statistics indicate that 57,7% of Romanians are overweight or obese [30]. Currently, the major causes of death in Romania are diet-related non-communicable diseases, such as cardiovascular diseases, stroke, cancer, and type 2 diabetes [31].

The purpose of this study is to examine the relationships between sociodemographic characteristics, HL, self-reported health and morbidity, and non-adherence to dietary recommendations and FL concepts (the connection between health and nutrition and nutrition information seeking) of a representative sample from the North-Western region of Romania. The results of this study could support the design of public health services in Romania by targeting specific groups that could benefit from food literacy programs and reaching those with inadequate food literacy-related abilities and dietary intake.

## 2. Materials and Methods

### 2.1. Study Design

We performed a secondary analysis on cross-sectional data gathered for the project named “Evaluating and enhancing the population’s knowledge in the North-West region of Romania regarding soy product types and the influence of nutrition on health.” The data set comprises information from 1715 participants residing in five counties within the North-Western region of Romania. This region covers 2 million individuals and is divided into five counties, which include 35 urban and 340 rural communities. A representative stratified random sample from this region was selected using a systematic random sampling method. A proportional number of households were systematically randomly selected according to the size of urban and rural areas from the Romanian counties of Bihor, Sălaj, Bistrița-Năsăud, Maramureș, and Cluj. In the five regions, 15 urban and 28 rural communities were selected, then polling sites, and then the starting streets at each polling site. After the initial starting point, every fifth address was selected to which one questionnaire was filled out until the required questionnaires were collected for that starting place. Thus, 1715 households were included in this study between March and November 2019. The survey questions were read by trained field technicians who completed the questionnaire using the Survey Monkey platform on mobile devices, ensuring consistent administration. The eligibility criteria for participants were: 18 years or above, Romanian residency and language, no clear sign of psychological or learning disabilities, and willingness to answer the survey. Additionally, data obtained from 143 participants were excluded from the current analysis due to incomplete responses regarding dietary intake, health literacy, and food literacy domains.

### 2.2. Questionnaire

The questionnaire is part of a more extensive study that assessed general and sociodemographic characteristics of the sample, questions regarding self-rated health and morbidity, dietary behaviour, health literacy, food literacy concepts, interest in healthy eating, perceptions of being informed regarding health aspects, the use and understanding of nutrition labels, and general notions about sugar and soy consumption. In this cross-sectional study, we utilised questions from previously validated questionnaires in other settings as the basis for our survey instrument. The items selected from previously developed instruments were methodically translated into Romanian and adapted following WHO guidelines, involving steps like translation, expert panel review, back-translation, pre-testing, cognitive debriefing, and final consensus [32]. The current study presents adherence to dietary recommendations and FL concepts in relation to sociodemographic characteristics, health literacy and self-reported health features. The general and sociodemographic questions include gender, residence, age, educational attainment, job status, marital status, and parental status. The questionnaire was pilot tested on a convenience sample of 100 persons for content validity, comprehensiveness, and reliability before data collection started.

### 2.3. Measures

#### 2.3.1. Adherence to Dietary Recommendations

Participants reported their food consumption behaviour using a food frequency questionnaire comprising 18 variables that measured their consumption of starchy products, fruits, nuts and seeds, vegetables, milk and dairy products, meat, fish and seafood, eggs, oil and fats, and water. The initial question, “How often do you eat the following foods?”, queried participants about the frequency of their regular consumption of each food group in the last month individually (7-point scale: “more than six times a day”, “2–5 times a day”, “once a day”, “3–5 times a week”, “1–2 times per week”, “2–3 times a month”, and “never/less than once a month”). The instrument was based on pre-existing validated food frequency questionnaires adapted and translated for the study [33,34]. During the administration of the food frequency questionnaires, the field technicians presented examples of portion sizes for each food group. These portion sizes were based on the Food-Based Dietary Guidelines reported by the Romanian Nutrition Society [35]. The technicians provided visual references to assist participants in accurately estimating their food portion sizes for each specific food group. In the pilot phase of the study, the internal consistency of the questionnaire, as assessed by Cronbach’s alpha, was found to be high (α = 0.917), indicating strong reliability of the instrument for our sample. For our analysis, we used the National Food-Based Dietary Guidelines [35] to compute the variables of adherence to fruit and vegetables, fish and seafood, and water recommendations. For the number of fruit and vegetable servings a day, the recommended cut-off was more than five times a day. The fish and seafood intake had a cut-off of more than 1–2 times per week, and the cut-off for water intake was more than six glasses per day.

#### 2.3.2. Food Literacy Concepts

The basis for our survey instrument was the Short Food Literacy Questionnaire (SFLQ) for adults, a validated questionnaire for measuring food literacy in an adult population developed by Krause et al. [36]. We adapted and translated three questions from the SFLQ that were most relevant to the aim of the study, namely: (1) “When I have questions on healthy nutrition, I know where I can find information on this issue”; (2) “How easy is it for you to evaluate the longer-term impact of your dietary habits on your health?”; (3) “How easy is it for you to evaluate if a specific food is relevant for a healthy diet?”. The first question measures the ability to acquire information about food, food preparation, and the influence of nutrition on health (functional food literacy). The last two questions evaluate critical food literacy domains: (2) the ability to understand the connection between nutrition and health, (3) to assess whether a food contributes to healthy nutrition and to distinguish between healthy and less healthy options, (4) to determine if a food contributes to healthy nutrition. The responses were measured on a Likert scale from 1 (“very difficult”) to 4 (“very easy”). For our analysis, the questions were dichotomised into “difficult” (scored 1) and “easy” (scored 0). During the pilot study phase, the three items were administered, demonstrating satisfactory internal consistency as evidenced by a Cronbach’s alpha of 0.769.

#### 2.3.3. Health Literacy

Health literacy was assessed using the Romanian version of the European Health Literacy Survey Questionnaire, a short version with 16 items (HLS-EU-Q16). A separate article was previously published containing the results on the validation of the HLS-EU-Q16 questionnaire and exploring HL predictors in this sample from the North-Western region of Romania [37]. The questionnaire demonstrated good internal consistency during pretesting with a Cronbach’s alpha of 0.813. In the current data analysis of the HLS-EU-Q16 questionnaire, the total scores obtained were treated as continuous data.

#### 2.3.4. Self-Perceived General Health and Morbidity

Self-perceived general health and morbidity were assessed using translated questions from the European Health Interview Survey [38]: (1) “How is your health in general?”, which was rated on a 5-point Likert scale from 1 = very good to 5 = very bad, and (2) “Do you have any longstanding illness or health problem? [By longstanding, I mean illnesses or health problems that have lasted, or are expected to last, for six months or more.]” with the following response possibilities “Yes, more than one,” “Yes, one longstanding illness,” and “No”. These were recoded as moderate/good general health (1–3) and poor health (4–5) and, respectively, yes (1–2) and no (3). The two self-reported indicators represent reliable and valid instruments to measure health in general and ill health [39,40,41,42].

### 2.4. Statistical Analysis

All statistical analyses were performed using SPSS Software (version 29; MacOS). Data were reported as frequencies and percentages for categorical variables and as means and standard deviations for continuous variables. Univariate and multivariate logistic regression models were conducted to evaluate the relationship between dependent variables, including sociodemographic characteristics (gender, residence, age, educational attainment, job status, marital status, and parental status), self-reported health features (self-perceived general health and morbidity), and health literacy, and the independent variables, namely non-adherence to dietary recommendations and low self-rated food literacy abilities. A Pearson correlation analysis was conducted to examine the relationships between non-adherence to dietary recommendations, low self-rated food literacy domains, health literacy, and self-reported health and morbidity. The statistical significance threshold chosen was *p* < 0.05.

## 3. Results

### 3.1. Participant Profile

Out of 1715 participants who responded to the survey (response rate equal to 72.63%), 1572 participants were included in the current statistical analysis. We excluded respondents who did not answer questions regarding health literacy, food literacy, and items regarding food intake. The sociodemographic features of the participants are presented in Table 1. Most of the sample were females (62.0%), and most lived in rural areas (53.0%). The average age was 52.94 years (DS = 16.83). Regarding educational attainment, respondents’ most common form of education was high school (44.5%), followed by university studies (21.0%). A small percentage of the sample (0.5%) had no formal education. Most of the sample was economically inactive, with 55.2% of participants identifying as students, on pension/social welfare, or homemakers. Most participants included in the study were married (68.6%) and reported having children (81.2%).

The levels of health literacy in the sample tested are considered unsatisfactory, with 42.9% of the participants having sufficient HL levels and only 16.3% having excellent health literacy levels. In our sample, most participants reported having moderate to good health (85.3%), with a smaller group reporting poor health (14.7%). Regarding longstanding illnesses, a slight majority of participants (53.3%) indicated they did not have any. In contrast, nearly half of the participants (46.7%) reported having at least one longstanding illness.

### 3.2. Factors Associated with Non-Adherence to Dietary Recommendations

Table 2 shows factors associated with non-adherence to dietary guidelines for daily fruit and vegetable consumption, weekly fish and seafood consumption, and daily water consumption. The overall percentage of participants not adhering to more than five portions of fruit and vegetables per day was 83.5%. The level of non-adherence to the recommended amount of fruit and vegetables was significantly lower for participants unemployed and looking for a job (OR = 0.40, 95% CI: 0.20–0.80), those retired or on social welfare (OR = 0.53, 95% CI: 0.40–0.72), elderly (OR = 0.98, 95% CI: 0.98–0.99), and who self-reported living with at least one chronic condition (OR = 0.69, 95% CI: 0.53–0.91). The dietary recommendation for weekly fish and seafood consumption was not followed by 61.3% of the sample. There was a significant difference between non-adherence to the weekly recommended amount of fish and seafood intake and gender, residence, educational attainment, marital status, job status, and health literacy. Fish and seafood consumption was lower among rural residents (OR = 1.49, 95% CI: 1.22–1.83), individuals who did not complete secondary education (OR = 2.07, 95% CI: 1.58–2.70), who were formerly married (OR = 1.50, 95% CI: 1.14–1.96), who have retired or receive social welfare (OR = 1.34, 95% CI: 1.08–1.66), and students (OR = 2.13, 95% CI: 1.18–3.85). Non-adherence to fish and seafood dietary recommendations was negatively associated with a higher health literacy score (OR = 0.98, 95% CI: 0.96–0.99) and being male (OR = 0.75, 95% CI: 0.61–0.93). In the case of water consumption, 67.9% of the sample reported inadequate intake. There was a significant negative association between inadequate intake and the health literacy score (OR = 0.98, 95% CI: 0.96–0.99) and a positive association with older age (OR = 1.00, 95% CI: 1.00–1.01), low educational attainment (OR = 1.32, 95% CI: 1.01–1.72), and poor self-rated health (OR = 1.75, 95% CI: 1.26–2.43).

In the multivariable model of factors influencing dietary habits, specific variables were found to significantly affect the consumption of fruits and vegetables, fish and seafood, and water intake.

Employment status emerged as a key factor for those consuming fewer than 5 portions of fruit and vegetables per day. Those unemployed and searching for work were 61.8% less likely (aOR: 0.38, 95% CI: 0.18–0.78) to meet this consumption level compared to employed individuals. Similarly, retirees or those on social welfare were 52.4% less likely (aOR: 0.476, 95% CI: 0.30–0.74) to consume the recommended portions of fruits and vegetables.

In terms of fish and seafood consumption, place of residence and education level significantly influenced the likelihood of eating less than one portion per week. Residents of rural areas were 1.47 times more likely (aOR: 1.47, 95% CI: 1.18–1.82) to fall below this consumption level compared to their urban counterparts. Individuals who had not completed secondary education were 62% more likely (aOR: 1.62, 95% CI: 1.19–2.19) to consume less fish and seafood.

For those consuming less than 1.5 litres of water daily, self-reported health status and the presence of longstanding illness were critical. Individuals with poor health were 81.3% more likely (aOR: 1.81, 95% CI: 1.26–2.59) to consume less water. Conversely, those with at least one longstanding illness were 28.2% less likely (aOR: 0.71, 95% CI: 0.55–0.92) to fall short of this water consumption level.

Other factors such as gender, marital status, parental status, age, and health literacy did not exhibit significant associations in these multivariate models. While potentially impactful, they did not meet the statistical significance threshold in this analysis.

### 3.3. Factors Associated with Poor Food Literacy-Related Abilities

The results of the evaluated food literacy concepts are more encouraging, with most participants reporting an adequate ability to understand the connection between nutrition and health (89.1%), to distinguish between healthy and less healthy options (84.4%), and to acquire information about nutrition (75.6%). Table 3 summarises the results concerning sociodemographic and health-related factors associated with food literacy.

An inadequate understanding of the connection between nutrition and health was positively associated with individuals living in a rural setting (OR = 1.60, 95% CI: 1.13–2.26) who had not finished secondary education (OR = 2.64, 95% CI: 1.85–3.75), with those formerly married compared to those currently married (OR = 1.68, 95% CI: 1.15–2.44), having children (OR = 1.86, 95% CI: 1.12–3.10), with those retired or on social welfare compared to employed (OR = 1.59, 95% CI:1.11–2.27), with older age (OR = 1.02, 95% CI: 1.01–1.03) and with poor self-rated health (OR = 1.59, 95% CI: 1.05–2.41). A higher health literacy score was negatively associated with a low understanding of the connection between nutrition and health (OR = 0.84, 95% CI: 0.82–0.87), as well as with those who never married compared to coupled individuals (OR = 0.46, 95% CI: 0.22–0.98).

A low ability to distinguish between healthy and less healthy options was more frequent in men (OR = 1.76, 95% CI: 1.33–2.32), elderly (OR = 1.00, 95% CI: 1.00–1.01) and those with poor-self rated health (OR = 1.67, 95% CI: 1.18–2.38). High health literacy (OR = 0.89, 95% CI: 0.87–0.91) reported a negative association with a low ability to distinguish between healthy and less healthy options.

A low ability to acquire nutrition information was significantly more frequent in those of older age (OR = 1.03, 95% CI: 1.02–1.03), rural residence (OR = 1.37, 95% CI: 1.08–1.74), who had not finished high school (OR = 2.68, 95% CI:2.06–3.49), were formerly married (OR = 2.17, 95% CI: 1.65–2.86), have children (OR = 1.55, 95% CI: 1.12–2.15), and those who are retired or on social welfare compared to employed (OR= 2.27, 95% CI: 1.76–2.93). In terms of self-reported health features, living with at least one longstanding illness (OR = 1.69, 95% CI: 1.34–2.14) and poor self-rated health (OR = 2.96, 95% CI: 2.21–3.97) were positively associated with a low ability to acquire nutrition information. Individuals with high health literacy (OR = 0.85, 95% CI: 0.83–0.87) and those who never married (OR = 0.59, 95% CI: 0.37–0.94) were more likely to self-report that they know where to obtain information on healthy nutrition.

In the multivariate model for assessing factors associated with a low understanding of nutrition and health, a low ability to distinguish between healthy and less healthy options, and a low ability to acquire information about nutrition, several significant variables were identified across the three dependent variables.

Health literacy was the most prevalent, affecting all three dependent variables. Each unit increase in the scale significantly reduced the odds of low understanding of nutrition and health by 15% (aOR: 0.85, 95% CI: 0.82–0.88), low ability to distinguish between healthy and less healthy options by 11% (aOR: 0.89, 95% CI: 0.87–0.91), and low ability to acquire information about nutrition by 13% (aOR: 0.87, 95% CI: 0.84–0.89).

Being female was significantly associated with being 1.75 times more likely to have a low ability to distinguish between healthy and less healthy options (aOR: 1.75, 95% CI: 1.27–2.40). Residence in a rural area was associated with a 38% lower likelihood of having a low ability to distinguish between healthy and less healthy options (aOR: 0.62, 95% CI: 0.45–0.85).

The parental status also significantly affected the ability to distinguish between healthy and less healthy options, with parents being two times more likely to report a low ability (aOR: 1.94, 95% CI: 1.08–3.48).

Being separated, divorced, or widowed significantly increased the likelihood of having a low ability to acquire information about nutrition by 59% (aOR: 1.59, 95% CI: 1.12–2.26). Having poor health status was associated with an 82% higher likelihood of having a low ability to acquire nutrition information (aOR: 1.82, 95% CI: 1.26–2.62). Finally, each additional year of age increased the likelihood of having a low ability to acquire nutrition information by 1.5% (aOR: 1.015, 95% CI: 1.00–1.03).

The following variables did not show a statistically significant association with any of the three dependent variables: education level, employment status, and the existence of a medical condition.

### 3.4. The Association between Non-Adherence to Dietary Recommendations, Self-Reported Health Features, Health Literacy, and Food Literacy Concepts

A Pearson correlation examined the relationship between non-adherence to dietary recommendations, healthy literacy, self-reported health and morbidity, and low self-rating of food literacy abilities (Table 4). The relationship between low self-rating of abilities regarding food literacy was statistically significant, positive, and weak in strength between all domains, except for the ability to acquire nutrition information and distinguish healthy and less healthy options, which was moderate in power. Non-adherence to the dietary guidelines regarding the intake of fish and seafood products was positively associated with a low ability to acquire nutrition information. Inadequate hydration behaviour was positively associated with low trust in nutritional information from media, non-adherence to fruit and vegetable recommendations, as well as with fish and seafood intake recommendations. Poor self-rated health showed a significant positive correlation with low self-rating of three of the abilities regarding food literacy (except for trust in nutritional information from media) and low water intake. Living with a longstanding illness showed a significant positive correlation with poor self-rated health and a low ability to acquire information about nutrition, while a negative correlation with a low intake of fruit and vegetables. The relationship between health literacy and the other variables was statistically significant and negative, except for fruit and vegetable intake. The relationships of health literacy ranged from weak to moderate in strength for all the variables examined.

## 4. Discussion

This study contributes to the existing knowledge by providing a comprehensive analysis of the relationship between food literacy, adherence to dietary recommendations, sociodemographic characteristics, and health-related features among adults in the North-Western region of Romania. The representative sample in our study is predominantly consistent with the data reported by the National Institute of Statistics in 2019, with the proportion of individuals living in urban (46.8%) and rural (53.0%) areas in our sample closely resembling that of the North-Western population in 2019 (52.41% urban and 47.59% rural). Similarly, when considering the economically active population in our sample (47.4%), the percentage is near the rate recorded for Romania in 2019 (47.6%). The economically inactive population in our sample (52.3%), with 42.9% being pensioners or social welfare recipients, aligns with the national statistics for the economically inactive population aged 15 and over (50.78%) and the average number of pensioners in 2019 (35.63%) [43].

Regarding dietary intake, the European Health Interview Survey findings showed that only 2% of the Romanian population eats at least five portions of fruit and vegetables daily [44,45]. This differs from the findings presented here, where the overall percentage of Romanian participants adhering to more than five portions of fruit and vegetables per day was 16.5%. In this study, we found that older individuals, those who are retired, receiving social welfare, or unemployed were less likely to deviate from the recommended fruit and vegetable dietary guidelines. This study brings new insights to the understanding of fruit and vegetable consumption patterns among different sociodemographic groups in Romania. Our research shows that older individuals, those retired or on social welfare, and the unemployed were less likely to deviate from the recommended fruit and vegetable dietary guidelines. Interestingly, our findings contradict the notion of a clear socioeconomic and educational gradient in fruit and vegetable consumption often reported in the literature, which found lower consumption rates among the low-income-education groups compared to the high-income-education groups [46,47,48,49]. Contrary to several previous studies [49,50], we found no gender difference in fruit and vegetable intake in the Romanian sample. The discrepancy between these findings may be attributed to a variety of factors not fully explored in our study, such as cultural traditions, dietary habits, or the relative affordability and accessibility of fresh produce in Romania compared to other regions. This suggests that gender-related dietary habits may vary widely by geographical and cultural context, reinforcing the importance of local data in public health research and planning. This is a tendency in the north and west regions and has a reverse tendency in the south and east regions of Europe [51]. The current results seem consistent with other research, which found that in areas where the availability and affordability of fruits and vegetables are higher, lower socioeconomic categories tend to consume more fruits and vegetables than individuals with a higher socio-economical rank [51,52]. Roos et al. suggest that individuals from lower socioeconomic classes from the south and east European regions may have increased accessibility to more affordable vegetables and fruits and are more likely to grow or procure vegetables from non-commercial channels [51].

Another important finding was that 38.7% of the sample reported fish and seafood intake at least once per week. These results support previously published studies, which showed that between 39–41% of Romanians consume fish and seafood at least once per week [53]. Fish and seafood consumption was lower among men, those from rural settings, those who did not complete secondary education, were formerly married, retired, or on social welfare, and students. This is in accordance with previously published data, where higher socioeconomic status (e.g., higher income, higher educational attainment) is associated with higher fish consumption frequency [54,55]. This finding is consistent with previously reported data on fish and seafood consumption, which shows that women, compared to men, have a higher intake of fish [54,56,57,58]. Regarding water intake, only 32.1% of the sample consumed at least six glasses of water per day. Older individuals reported inadequate fluid intake more frequently. No previous data regarding water intake was recorded in Romania, although data is available for other European countries [59,60,61]. Earlier studies with data from European countries have demonstrated that the percentage of individuals with a markedly low fluid intake (<1500 mL) was found to be between 24–42% for women based on different age groups and between 33–43% for men, respectively [59]. The current findings align with previous research in demonstrating the interconnectedness of water intake with age, health literacy, educational attainment, and self-rated health. Our finding of a negative association between inadequate water intake and high health literacy scores is supported by previous studies [62,63], highlighting the critical role of health literacy in hydration, particularly among older adults. The positive association we found between inadequate water intake and older age aligns with previous work [64,65,66], which emphasises the decrease in water consumption and thirst sensation with advancing age.

Our results also suggest the influence of education level on water intake, reinforcing the idea that socioeconomic factors, including education, play a significant role in healthful behaviours, such as adequate hydration [65,67,68]. Finally, the positive association we found between inadequate water intake and poor self-rated health is supported by the literature [64,69], which suggests that low water consumption might coexist with other unhealthy behaviours and attitudes and is associated with poorer self-rated health status. Overall, the current study contributes to a growing body of evidence suggesting the need for a comprehensive approach to promoting adequate water intake, considering a variety of factors such as age, health literacy, education level, and self-rated health.

Existing knowledge about food literacy abilities and what influences them needs to be improved. This often leads to assumptions being made about the most suitable groups for targeted health programs [70]. This study sheds new light on these issues, offering a unique view into distinct food literacy abilities, dietary habits, and participants’ demographic profiles that would benefit from a food and health literacy program in Romania. The findings on measuring health literacy in Romania indicated that gender and education demonstrated a positive association with health literacy, while age and self-reported health showed a negative association with health literacy [37]. Even though government programs often focus on the needs of low-income and disadvantaged populations [71,72], the insights from this study deepen our understanding of food literacy-related aspects, which could benefit a wider range of individuals.

Our study also explored the influence of various sociodemographic factors on food literacy-related abilities. It was noted that men reported a lower capacity to distinguish between healthy and less healthy options. This echoes the recommendation for tailoring food literacy programs to demographic factors such as gender [15]. This is consistent with findings by Lee, who observed that women generally exhibit more positive attitudes toward food and good eating habits, which was attributed to sociocultural factors and their traditionally assigned role in preparing and cooking food [73]. The research conducted by Krause et al. [36] and Sponslee et al. [74] corroborated that females display superior levels of food and health literacy compared to their male counterparts and are more likely to incorporate healthy dietary practices into their daily routines.

Further, we found that individuals from lower socioeconomic backgrounds, such as those without a high school diploma, retired or on social welfare, and formerly married, had a lower ability to understand the connection between nutrition and health and to acquire nutrition information. These findings align with previous research, which indicates that people with lower socioeconomic status have lower food literacy levels [15].

Other researchers employing representative samples from the population underscore that specific demographic groups—such as males, younger adults, and those with limited education—may yield more efficacious outcomes from cooking and food skills-focused programs [75,76]. The current results regarding the fact that older individuals and those that are retired or on social welfare had issues with the ability to understand the connection between nutrition and health, distinguish between healthy and less healthy options, and acquire information about nutrition, may be explained by the national economic and social development context, in which the growing number of retired individuals exceeded the number of employees in the economy. The current social system for older people needs to provide a decent household income. Older persons from Romania are the social category subjected to the highest injustices in the market economy [77,78].

Our findings are in line with a study which investigated nutrition information-seeking behaviour in five European countries, and reported notable disparities in seeking nutrition information. It is primarily men, individuals with low levels of education, low income, and poor health among the surveyed populations who profess not to seek nutritional information actively. As such, these population groups should be prioritised for targeted nutrition information education [79]. A recent study further analysed the segmentation of consumers based on nutrition information-seeking behaviour, and, in the study, a category of consumers termed ‘uninterested consumers’ was characterised by a low level of food literacy, a tendency to prioritise cost over nutrition, and a marked difficulty in engagement. Predominantly older males with the least education and food literacy among the groups studied made up this category. These individuals tended to value the affordability of food more than its nutritional content, resulting in a lower frequency of consumption of healthful foods. The findings highlight the ineffectiveness of merely providing information to this group, emphasising the necessity for a more nuanced approach [80]. When examining the eating habits of Romanian, a previous study found that they tend to adopt unhealthy eating habits, consuming foods high in saturated fats, sugar, and additives [29]. These habits reflect the influence of highly industrialized societies and the prevalence of unbalanced food products. However, there is a positive trend emerging among Romanian consumers towards healthier and more sustainable food options, characterised by a preference for foods with a low degree of processing. Our study contributes by adding context to the need for tailored food literacy programs and targeted nutrition information education for specific population groups.

Individuals with poor self-rated health and inadequate health literacy reported low food literacy levels, reaffirming that insufficient health literacy can be a barrier to understanding nutrition and healthy food practices [17,81]. Living with a longstanding illness was positively correlated with poor self-rated health and a low ability to acquire nutritional information in our sample. This aligns with previous research that suggests healthy individuals tend to have higher health and food literacy levels than individuals with chronic diseases. This underlines the importance of health and food literacy in disease self-management and indicates a potential area of intervention for improving health outcomes [79].

This study was a comprehensive cross-sectional study with large sample size. However, due to this design, the findings should be interpreted cautiously regarding temporality or causation. The sample was randomly selected, so it is representative of the North-Western region of Romania. However, not all participants approached desired to be part of the study, which might represent self-selection bias, even with the control measures in place such as randomised polling sites, streets and using a specific data collection pace. It is possible that individuals who did not complete the evaluation were from culturally and linguistically diverse groups who were possibly the least health and nutrition literate in a Romanian context.

The overrepresentation of women in the study sample, potentially due to the timing of data collection during working hours, may have introduced some bias. Despite this, our sample is not drastically misaligned with the general population of the North Western region of Romania, where women made up 51.70% in 2019, according to the National Institute of Statistics [43]. Nonetheless, the potential overrepresentation of women might slightly skew the reflection of the targeted population in this study. While the data collection period (March to November 2019) was designed to minimise the impact of potential seasonal variation in diet, we acknowledge that the possibility of seasonal effects on dietary habits cannot be completely excluded. In addition, this study examined multiple factors affecting healthy nutrition behaviours, attitudes, and knowledge. Considering that the data was self-reported and obtained using subjective questionnaires, it may be subject to a social desirability bias. It might reflect self-confidence rather than actual abilities and knowledge. The findings about adherence to dietary guidelines and food literacy-related skills were only partially comprehensive due to constraints such as the extensive length of the questionnaire, cognitive load, and reading level considerations, which could potentially burden respondents. The questions that were incorporated were deemed significant for this population based on a review of the limited existing literature on the subject and the consensus of the research team. Therefore, future studies are suggested to obtain more information, including the exploration of more food literacy domains and determinants that still need to be tested (e.g., disease and anthropometric data).

Within the field of public health nutrition in Romania, this study contributes to the limited body of research in the field and highlights a deeper understanding of the importance of improving healthy eating behaviours and abilities in disadvantaged Romanians. These results are important to governmental authorities leading public health and health professional organisations for the development of effective nutrition programs and strategies for addressing specific needs. Public health nutrition campaigns and educational programs may aim to improve consumers’ abilities regarding healthy eating to positively influence their health literacy and knowledge, adherence to dietary recommendations, and general health. The success of different approaches and tools that may be used to educate consumers about the consequences of their dietary choices for their health depends on the consumers’ interests in healthy eating and favourable attitudes towards these concepts. Educating consumers about the consequences of their dietary choices for their health may raise awareness and interest in health and improve the subjective significance of this issue when making food choices.

## 5. Conclusions

The present research aimed to examine adherence to dietary recommendations, food literacy-related abilities, and their determinants in a representative sample from a region in Romania. These aspects need to be adequately investigated in Romania and Eastern Europe. The results indicate an independent association with health literacy, self-rated health, dietary behaviour, and food literacy. Socioeconomic inequalities (lower education attainment, individuals living in rural areas, older age categories, and job status) play a significant role in dietary behaviours and food literacy. Recognising and addressing problems of low food literacy abilities and non-adherence to dietary recommendations in participants could facilitate the reduction of possible long-term negative health outcomes.

## Figures and Tables

**Table 1:** Sample description (*n* = 1572).

Variables		*n*	%
Gender	Female	974	62.0
	Male	598	38.0
Age (years)	18–29	169	10.8
	30–39	199	12.7
	40–49	271	17.2
	50–59	307	19.5
	60–69	356	22.6
	70 years and older	270	17.2
Residence	Urban	736	46.8
	Rural	833	53.0
	Missing	3	0.2
Educational attainment	Without formal education	8	0.5
	Elementary school	70	4.5
	Middle school	257	16.3
	High school/Professional school	699	44.5
	Post-secondary school	155	9.9
	University	330	21.0
	Missing/Not wanting to respond	53	3.4
Job status	Worker	699	44.5
	Job-seeking unemployed	46	2.9
	Pension or on social welfare	675	42.9
	Student	61	3.9
	Homemaker	87	5.5
	Missing/Not wanting to respond	4	0.3
Parental status	Parent	1276	81.2
	Non-parent	296	18.8
Marital status	Currently married	1079	68.6
	Never married	203	12.9
	Widowed	226	14.4
	Separated or divorced	45	2.9
	Cohabiting	15	1.0
	Missing/Not wanting to respond	4	0.3

**Table 2:** Factors associated with non-adherence to dietary recommendations.

Variables		Fruit & Vegetables <5 Portions/Day		Fish & Seafood <1 Portion/Week		Water <1.5 Litters/Day	
		*n* (%)	OR (95% CI)	*n* (%)	OR (95% CI)	*n* (%)	OR (95% CI)
Gender	Female	804 (82.5)	Ref.	621 (63.8)	Ref.	662 (68.0)	Ref.
	Male	508 (84.9)	1.19 (0.90–1.57)	342 (57.2)	0.75 (0.61–0.93) **	406 (67.9)	0.99 (0.80, 1.24)
Residence	Urban	626 (85.1)	Ref.	413 (56.1)	Ref.	509 (69.2)	Ref.
	Rural	684 (82.1)	1.24 (0.94, 1.62)	547 (65.7)	1.49 (1.22, 1.83) ***	557 (66.9)	0.90 (0.72, 1.11)
Educational attainment	Finished high school	996 (84.1)	Ref.	677 (57.2)	Ref.	781 (66.0)	Ref.
	Not finished high school	269 (80.3)	1.30 (0.95, 1.77)	246 (73.4)	2.07 (1.58, 2.70) ***	241 (71.9)	1.32 (1.01, 1.72) *
Marital status	Married/Cohabiting	917 (83.8)	Ref.	643 (58.8)	Ref.	738 (67.5)	Ref.
	Never married	147 (85.0)	1.09 (0.69, 1.70)	112 (64.7)	1.28 (0.92, 1.79)	117 (67.6)	1.00 (0.71, 1.42)
	Formerly married	248 (81.3)	0.84 (0.60, 1.16)	208 (68.2)	1.50 (1.14, 1.96) ***	213 (69.8)	1.11 (0.84, 1.47)
Parental status	Non-parent	252 (83.1)	Ref.	185 (62.5)	Ref.	196 (66.2)	Ref.
	Parent	1060 (85.1)	0.85 (0.60, 1.21)	778 (61.0)	1.06 (0.82, 1.38)	872 (68.3)	0.90 (0.69, 1.18)
Labour Market status	Worker	612 (87.6)	Ref.	397 (56.8)	Ref.	461 (66.0)	Ref.
	Job-seeking unemployed	34 (73.9)	0.40 (0.20, 0.80) **	30 (65.2)	1.42 (0.76, 2.66)	35 (76.1)	1.64 (0.82, 3.29)
	Retired/Social welfare	534 (79.1)	0.53 (0.40, 0.72) ***	431 (63.9)	1.34 (1.08, 1.66) **	475 (70.4)	1.22 (0.97, 1.53)
	Student	56 (91.8)	1.59 (0.62, 4.08)	45 (73.8)	2.13 (1.18, 3.85) *	43 (70.5)	1.23 (0.69, 2.18)
	Homemaker	74 (85.1)	0.80 (0.43, 1.52)	57 (65.5)	1.44 (0.90, 2.30)	50 (57.5)	0.69 (0.44, 1.09)
Age	Years (continuous)	−3.25 (−1.02, −5.49)	0.98 (0.98, 0.99) **	0.69 (−1.01, 2.40)	1.00 (0.99, 1.00)	1.96 (0.18, 3.75)	1.00 (1.00, 1.01) *
Health Literacy	Score (continuous)	−0.14 (−0.73, 1.01)	0.99 (0.97–1.01)	−0.72 (−1.38, −0.05)	0.98 (0.96, 0.99) *	−0.80 (−1.49, −0.10)	0.98 (0.96, 0.99) *
Self-rated health	Moderate/Good	1125 (83.9)	Ref.	812 (60.6)	Ref.	889 (66.3)	Ref.
	Poor	187 (81.0)	0.81 (0.57–1.16)	151 (65.4)	1.23 (0.91–1.64)	179 (77.5)	1.75 (1.26, 2.43) ***
Longstanding illnesses	No	716 (85.7)	Ref.	514 (61.6)	Ref.	570 (68.3)	Ref.
	Yes	591 (80.7)	0.69 (0.53–0.91) **	447 (61.1)	1.02 (0.83–1.25)	493 (67.3)	0.95 (0.77, 1.18)

* *p* < 0.05, ** *p* < 0.01, *** *p* < 0.001. Bold font denotes statistical significance. Statistical test: univariate logistic regression. Abbreviations: CI = confidence interval; OR = odds ratio. For continuous variables, values are presented as mean difference (MD) with a 95% confidence interval (95% CI) instead of *n* (%).

**Table 3:** Factors associated with low self-rated abilities regarding food literacy concepts.

Variables		Low Understanding of the Connection between Nutrition & Health		Low Ability to Distinguish between Healthy & Less Healthy Options		Low Ability to Acquire Information about Nutrition	
		*n* (%)	OR (95% CI)	*n* (%)	OR (95% CI)	*n* (%)	OR (95% CI)
Gender	Female	86 (8.9)	Ref.	118 (12.2)	Ref.	225 (23.1)	Ref.
	Male	69 (11.6)	1.35 (0.96, 1.89)	117 (19.6)	1.76 (1.33, 2.32) ***	144 (24.2)	1.05 (0.83, 1.34)
Residence	Urban	56 (7.6)	Ref.	123 (16.8)	Ref.	150 (20.4)	Ref.
	Rural	97 (11.7)	1.60 (1.13, 2.26) **	110 (13.2)	0.75 (0.57, 1.00)	217 (26.1)	1.37 (1.08, 1.74) **
Educational attainment	Finished high school	91 (7.7)	Ref.	166 (14.1)	Ref.	221 (18.7)	Ref.
	Incomplete high school	60 (18.1)	2.64 (1.85, 3.75) ***	58 (17.4)	1.28 (0.92, 1.78)	128 (38.2)	2.68 (2.06, 3.49) ***
Marital status	Married/Cohabiting	102 (9.4)	Ref.	159 (14.6)	Ref.	232 (21.3)	Ref.
	Never married	8 (4.6)	0.46 (0.22, 0.98) *	25 (14.5)	0.98 (0.62, 1.56)	24 (13.9)	0.59 (0.37–0.84) *
	Formerly married	45 (14.8)	1.68 (1.15, 2.44) **	51 (16.7)	1.17 (0.83, 1.65)	113 (37.0)	2.17 (1.65–2.86) ***
Parental status	Non-parent	18 (6.1)	Ref.	35 (11.8)	Ref.	52 (17.6)	Ref.
	Parent	137 (10.8)	1.86 (1.12, 3.10) *	200 (15.7)	1.39 (0.94, 2.04)	317 (24.9)	1.55 (1.12, 2.15) **
Labor Market status	Worker	56 (8.0)	Ref.	95 (13.6)	Ref.	118 (17.0)	Ref.
	Job-seeking unemployed	7 (15.2)	2.05 (0.87, 4.78)	8 (17.4)	1.33 (0.60, 2.94)	10 (21.7)	1.36 (0.65, 2.81)
	Retired/social welfare	82 (12.2)	1.59 (1.11, 2.27) *	116 (17.2)	1.32 (0.98, 1.77)	214 (31.7)	2.27 (1.76, 2.93) ***
	Student	1 (1.6)	0.19 (0.02, 1.40)	7 (11.5)	0.82 (0.36, 1.85)	5 (8.2)	0.43 (0.17, 1.11)
	Homemaker	8 (9.2)	1.15 (0.53, 2.51)	9 (10.3)	0.73 (0.35, 1.50)	22 (25.3)	1.65 (0.98, 2.79)
Age	Years (continuous)	6.09 (3.31, 8.87)	1.02 (1.01, 1.03) ***	2.45 (0.12, 4.79)	1.00 (1.00, 1.01) *	8.11 (6.19, 10.04)	1.03 (1.02, 1.03) ***
Health Literacy	Score (continuous)	−6.48 (−7.52, −5.44)	0.84 (0.82, 0.87) ***	−4.52 (−5.41, −3.64)	0.89 (0.87, 0.91) ***	−5.74 (−6.45, −5.02)	0.85 (0.83, 0.87) ***
Self-rated health	Moderate/Good	123 (9.2)	Ref.	186 (13.9)	Ref.	270 (20.2)	Ref.
	Poor	32 (13.9)	1.59 (1.05, 2.41) *	49 (21.3)	1.67 (1.18, 2.38) **	99 (42.9)	2.96 (2.21, 3.97) ***
Longstanding illnesses	No	75 (9.0)	Ref.	116 (13.9)	Ref.	160 (19.2)	Ref.
	Yes	80 (11.0)	0.79 (0.57, 1.11)	119 (16.3)	0.83 (0.62, 1.09)	209 (28.7)	1.69 (1.34, 2.14) **

* *p* < 0.05, ** *p* < 0.01, *** *p* < 0.001. Bold font denotes statistical significance. Statistical test: univariate logistic regression. Abbreviations: CI = confidence interval; OR = odds ratio. For continuous variables, values are presented as mean difference (MD) with a 95% confidence interval (95% CI) instead of *n* (%).

**Table 4:** Relationship between non-adherence to dietary recommendations, health-related aspects, and food literacy concepts.

Variables	*n* (%)	(1)	(2)	(3)	(4)	(5)	(6)	(7)	(8)
(1) Low understanding of the connection between nutrition and health	155 (9.9)	1							
(2) Low ability to distinguish between healthy and less healthy options	235 (14.9)	−0.257 **	1						
(3) Low ability to acquire information about nutrition	369 (23.5)	0.199 **	0.312 **	1					
(4) Intake of fruit and vegetables < 5 portions/day	1312 (83.5)	0.010	0.005	0.021	1				
(5) Intake of fish and seafood products < 1 portion/week	963 (61.3)	0.031	0.008	0.093 **	0.008	1			
(6) Intake of water < 1.5 litters/day	1068 (67.9)	0.008	0.044	0.043	0.109 **	0.089 **	1		
(7) Health literacy score	49.68 (6.58)	−0.271 **	−0.234 **	−0.361 **	−0.007	0.053 *	−0.067 *	1	
(8) Poor self-rated health	231 (14.7)	0.056 *	0.073 **	0.189 **	−0.028	0.035	0.085 **	−0.174 **	1
(9) Living with a longstanding illness	732 (46.6)	0.034	0.033	0.112 **	−0.067 **	−0.005	−0.010	−0.141 **	0.345 **

* Correlation is significant at the 0.01 level (2-tailed), ** Correlation is significant at the 0.001 level (2-tailed). Bold font denotes statistical significance. Statistical test: Pearson correlation. For the health literacy score, values are presented as mean (M) with standard deviation (SD) instead of *n* (%).

## Data Availability

The data that support the findings of this study are available from Babeș-Bolyai University. Data are available from the authors with the permission of Babeș-Bolyai University.
